# Laser Powder Bed Fusion of a Novel Crack-Free γ′ Phase-Strengthened Ni-Based Alloy

**DOI:** 10.3390/ma18020237

**Published:** 2025-01-08

**Authors:** Defan Wu, Jiafeng Feng, Yi Wang, Zhijie Wang, Meng Wu, Quanquan Han

**Affiliations:** 1 Shenzhen Research Institute of Shandong University, Shenzhen 518057, China; 2 Key Laboratory of High Efficiency and Clean Mechanical Manufacture of Ministry of Education, School of Mechanical Engineering, Shandong University, Jinan 250061, China; 3 School of Automotive Engineering, Weifang Vocational College, Weifang 262737, China

**Keywords:** laser powder bed fusion, Ni-based alloy, cracking suppression, microstructure, mechanical properties

## Abstract

Laser powder bed fusion (LPBF)-fabricated Ni-based alloys with high γ′ phase fractions generally suffer from cracking that limits their applications. This study presents SD247, a novel alloy that overcomes the challenge of cracking issues and exhibits superior mechanical properties after heat treatment. Compared to CM247LC, SD247 exhibited a lower cracking tendency due to alloying element modification. After heat treatment, SD247 features primary γ′ phases with an average diameter of 306 nm and secondary γ′ phases averaging 25 nm, with few Ta- and Ti-rich MC-type carbides. The columnar grain structure in as-built specimens largely disappeared, and the fraction of grains in soft orientations decreased by 12.8%. The microhardness and ultimate tensile strength increased by 30% and 20%, respectively. These findings demonstrate that the superior LPBF fabrication of Ni-based alloys with a high γ′ fraction can be achieved. Because of the excellent mechanical properties and acceptable cost, LPBF-fabricated SD247 shows great potential in aerospace hot-end components.

## 1. Introduction

CM247LC is a precipitation-strengthened Ni-based alloy renowned for its outstanding mechanical properties, oxidation resistance, and corrosion resistance at elevated temperatures, all of which make it a key material for high-performance components in the aerospace field and in gas turbine engines [1]. However, traditional manufacturing methods such as casting and forging struggle to meet the fabrication requirements of complex engine components. In contrast, laser powder bed fusion (LPBF) technology, one of the metal additive manufacturing (AM), enables the layer-by-layer fabrication of complex geometries directly from a 3D model, which has sparked a growing interest in the application of LPBF for CM247LC components [2].

The high (Al + Ti) content (>7.0 wt.%) and the unique metallurgical process involved in LPBF contribute to significant challenges in the fabrication of CM247LC alloys. Cracking susceptibility is the primary issue among these challenges [3]. Cracking mechanisms in Ni-based alloys include solidification cracking, liquation cracking, strain-age cracking (SAC), and ductility dip cracking (DDC) [4,5]. Recently, researchers have devoted considerable attention to mitigating cracking in Ni-based alloys with high (Al + Ti) content. The cracking-suppression methods primarily include four types, as described below.

*Process optimisation*: Process parameters significantly influence the solidification process and the stress states within the LPBF process, both of which, in turn, affect cracking behaviour [6]. Carter et al. first noted that despite a wide range of process parameter adjustments, all LPBF-fabricated CM247LC alloy samples exhibited severe cracking defects [7]. Both Adegoke et al. and Hu et al. demonstrated that achieving crack-free LPBF manufacturing of CM247LC alloy could not be accomplished only by adjusting the volume energy density [8,9]. Similar findings from studies on other precipitation-strengthened Ni-based alloys with high (Al + Ti) content suggest that the optimisation of process parameters alone is insufficient to fully eliminate cracking.

*Hot isostatic pressing (HIP)*: The HIP technique leverages the combined effects of elevated temperature and pressure to enhance material densification [10]. Xu et al. and Lee et al. employed HIP to eliminate cracks in LPBF-fabricated IN738 and CM247LC alloys and found that some mechanical properties of the alloys were impaired [11,12]. Wei et al. also reported that direct HIP treatment cannot effectively eliminate microcracks in LPBF-fabricated René 142 alloy [13]. Cracks healed by HIP might reinitiate during subsequent heat treatment due to γ′ phase precipitation in Ni-based alloys with a high γ′ phase fraction [11]. These findings highlight the significant limitations of using HIP as the sole method for crack elimination.

*Particle decoration*: Cracking in LPBF-fabricated Al-based alloys and solution-strengthened Ni-based alloys has been mitigated through the addition of reinforcing particles, although similar results have not been observed in alloys with high (Al + Ti) content [14,15]. Lv et al. reported that incorporating TiC particles in LPBF-fabricated CM247LC alloys inhibited cracking but compromised the alloy’s ductility [3]. Similarly, Bahadur et al. and Wei et al. both found that introducing nanoparticles did not achieve complete crack suppression in LPBF-fabricated IN738LC and René 104 alloys [16,17]. While particle decoration can refine grains, reducing stress concentration, it cannot fully address the residual stresses caused by γ′ phase precipitation in alloys with high γ′ fraction [15].

*Alloying element modification*: Ni-based alloys designed for traditional forming processes are often difficult to adapt to the unique metallurgical conditions of LPBF, resulting in severe cracking susceptibility [18]. Griffiths et al. demonstrated that removing Hf from CM247LC alloys reduced the freezing range (FR), leading to significant decreases in crack density after LPBF [4]. Similarly, other researchers achieved the nearly crack-free LPBF fabrication of IN738 alloy by adjusting the contents of Si, B, and Zr [19,20,21]. In another study, Tang et al. designed ABD-850AM and ABD-900AM alloys for LPBF based on thermodynamic calculations; both alloys exhibited good LPBF processability and favourable mechanical properties [22]. More recently, multi-objective optimisation, machine learning, and other advanced methods have successfully developed several alloys capable of crack-free LPBF fabrication [18,23,24].

In summary, process optimisation, HIP, and particle decoration have demonstrated limited effectiveness in suppressing cracking in LPBF-fabricated precipitation-strengthened Ni-based alloys with high (Al + Ti) content. In contrast, the modification of alloying elements has demonstrated significant potential for achieving crack-free LPBF fabrication.

This study develops an SD247, a novel alloy with tailored compositions (removal of Hf and reduction in Al, B, and Zr compared to CM247LC), enabling excellent LPBF processability. Together with customised heat treatment, the alloy achieves high-quality LPBF fabrication. This study also explores the LPBF processability of SD247, systematically investigating the microstructure, microhardness, and tensile properties of both as-built and heat-treated alloys.

## 2. Materials and Methods

### 2.1. Experimental Materials and LPBF Process

**Figure 1:**
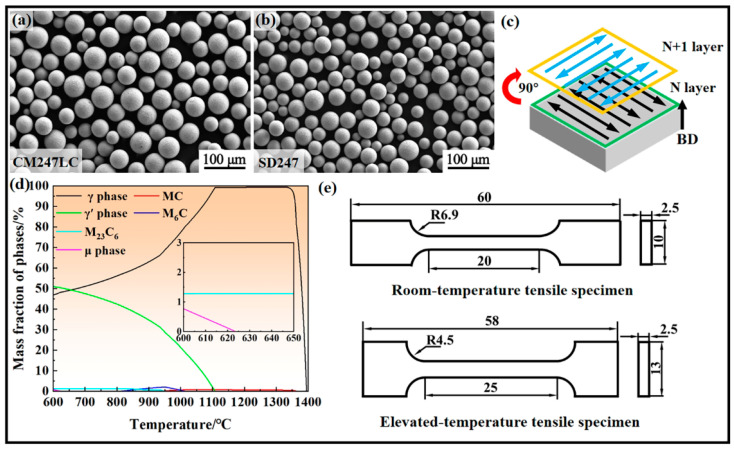
The morphology of (**a**) CM247LC and (**b**) SD247 powders; (**c**) scanning strategy; (**d**) phase diagram of SD247 in equilibrium solidification; (**e**) dimensions of tensile specimens.

The CM247LC and SD247 spherical powders used in this study were produced using the plasma rotating electrode process. Table 1 provides the chemical compositions determined by inductively coupled plasma optical emission spectrometry and infrared carbon–sulfur analysis. The morphologies of the two powders are shown in Figure 1a,b, respectively. Both powders exhibited high sphericity, indicating good flowability, and no satellite powders were observed. Their size distributions fell within the 15–53 μM range.

The LPBF process was performed using a Concept Laser Mlab Cusing 200R machine (GE, Colibrium Additive, Lichtenfels, Germany) equipped with a 200 W ytterbium fibre laser (IPG Photonics, Marlborough, MA, USA) under a protective atmosphere of 99.999% argon. Laser scanning speed (LSS) was selected as the key parameter for optimisation. The laser power in this study was fixed at 190 W to improve productivity. The 110 μm hatch spacing and 40 μm layer thickness were selected as guided by our previous studies because LSS is the dominant factor when laser power is fixed [5,15,25]. The LSS was varied between 400 mm/s and 1400 mm/s to explore the optimal processing window for SD247. To optimise heat dissipation and minimise residual stresses, a ‘stripe’ scanning strategy, with a 90° rotation between successive layers, was employed, as illustrated in Figure 1c. Two types of samples were fabricated: cubic samples (6 mm × 6 mm × 6 mm) for parameter optimisation, microstructure analysis, and microhardness testing, and block samples (65 mm × 15 mm × 2.5 mm) for tensile specimen fabrication.

### 2.2. Thermodynamic Calculations

The solidification paths were simulated using the Scheil–Gulliver module, and the phase diagram was calculated using the equilibrium solidification module of Thermo-Calc software (2023a) with the TCNI12 thermodynamic database. A temperature step of 1 K was applied in the thermodynamic calculations. The Scheil–Gulliver module operates under three key assumptions: (1) the distribution of elements at the solid/liquid interface is in equilibrium at all times, (2) elements in the liquid phase diffuse infinitely fast, and (3) no diffusion occurs in the solid phase [22]. These assumptions are generally valid during the rapid solidification processes of alloys, such as welding and AM. Numerous studies have also validated the model’s accuracy in predicting solidification paths and microstructure under the rapid heating and cooling conditions of LPBF [4,5,18,22,23]. The γ′ phase precipitation behaviour during the heat cycle in LPBF was modelled using the MOBNI6 kinetics database in conjunction with the TCNI12 thermodynamic database.

### 2.3. Heat Treatment

The heat treatment process was carried out in a tubular furnace under an argon atmosphere. A bimodal γ′ phase distribution and higher γ′ phase fraction may benefit the alloy’s mechanical properties [18,26]. Combined with our previous studies about phase precipitation behaviour, a heat treatment strategy for SD247 was developed to tailor microstructures, guided by the phase diagram for equilibrium solidification (Figure 1d) [5,18]. The treatment consisted of a solid solution step followed by a two-stage ageing process, as follows: (1) 1250 °C for 3 h with air cooling (AC), (2) 1080 °C for 4 h with AC, and (3) 650 °C for 48 h with AC.

### 2.4. Microstructure Characterisation and Mechanical Testing

Cubic samples were processed by wire cutting to obtain cross-sectional surfaces. To observe defects and microstructure, these surfaces were sequentially ground and polished using sandpaper (from 180# to 2500#), diamond suspension, and silicon dioxide polishing liquid. After polishing, as-built specimens were etched by a mixed solution of CuCl_2_, HCl, and C_2_H_5_OH (1:5:5) to examine the cellular structure, and a 15% oxalic acid solution at 3.3 V was used to reveal carbides and the γ′ phase in heat-treated specimens. Defects such as microcracks, keyholes, pores, and lack of fusion (LOF) were examined using an optical microscope (OM) to assess the LPBF processability of the alloys. Microcracks were defined as cracks with a width ≥ 2.5 μM and a length ≥ 30 μM, observable under a 50× optical lens in this study [15]. Crack-free fabrication was defined as the absence of cracks in six randomly selected areas (totalling approximately 8 mm^2^) on the polished surface of each sample. The microstructure and tensile fracture surfaces were analysed with a Gemini 500 (Carl Zeiss, Oberkochen, Germany) scanning electron microscope (SEM) equipped with electron backscatter diffraction (EBSD) and energy-dispersive X-ray spectroscopy (EDS). The EBSD step size was 1 μM, with a mapping area of 800 μM × 800 μM for each specimen. All OM, SEM, and EBSD analyses were performed on the side surface parallel to the building direction.

Room temperature (25 °C) microhardness was measured using an HVS-1000a microhardness tester (Huayuzongxin, Yantai, China), with a load of 200 gf applied for 10 s. The dimensions of the room temperature and elevated temperature (1100 °C) tensile specimens are shown in Figure 1e. Room temperature tensile tests were conducted using a Zwick 250 (Zwick, Zwick Roell, Ulm, Germany) universal testing machine at a strain rate of 0.04 min^−1^, while elevated temperature tensile tests were performed on a QJ212 tensile machine (Qingji, Shanghai, China) under an air atmosphere at a strain rate of 0.1 min^−1^. In all tensile tests, the loading direction was perpendicular to the building direction. The ultimate tensile strength (UTS), yield strength (YS) of SD247, and elongation (EL) were determined as the average of three tensile test results. The original gauge lengths of the room temperature and elevated temperature tensile specimens were 20 mm and 25 mm, respectively.

## 3. Results and Discussion

### 3.1. Overview of Processability

**Figure 2:**
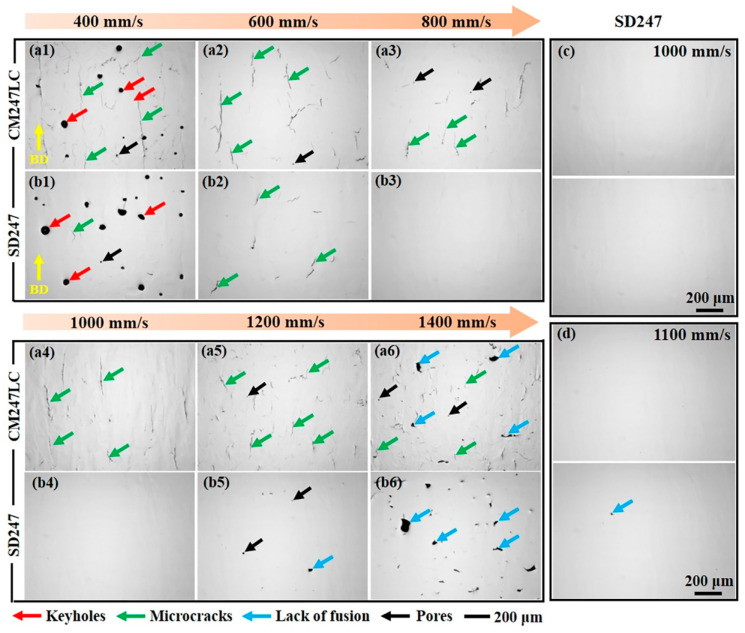
Optical microscopy (OM) images of laser powder bed fusion (LPBF) fabricated (**a1**–**a6**) CM247LC and (**b1**–**b6**) SD247 alloys at different laser scanning speeds (LSSs); OM images of LPBF-fabricated SD247 at (**c**) 1000 mm/s and (**d**) 1100 mm/s.

OM images of LPBF-fabricated CM247LC at various LSSs are shown in Figure 2(a1–a6). As the figure shows, defects were present in all samples, though the types of defects showed minimal variation. At lower LSSs, the predominant defects were keyholes and microcracks, both of which resulted from an unstable melt pool (Figure 2(a1)). At higher LSSs, the defects primarily consisted of LOF and microcracks, which likely occurred because of the reduced laser energy density (Figure 2(a6)). Notably, microcracks were observed across all LSSs, highlighting the severe cracking susceptibility of LPBF-fabricated CM247LC.

For SD247 alloy, significant keyholes and LOF defects were observed at 400 mm/s and 1400 mm/s, respectively, as shown in Figure 2(b1,b6)). A few LOF defects were also observed at 1200 mm/s (Figure 2(b5)). Microcracks were only detected at relatively low LSSs of 400 mm/s and 600 mm/s, which may have been related to higher residual stress levels at lower speeds [15]. Further analysis confirmed that no microcracks were present in six randomly selected areas on the surfaces of samples fabricated at 800 mm/s and 1000 mm/s. To assess process stability and determine the optimal LSS, two additional cubic samples were fabricated at 1000 mm/s and 1100 mm/s, with the corresponding OM images shown in Figure 2c,d. The sample fabricated at 1000 mm/s exhibited superior fabrication quality compared to the 1100 mm/s sample. Based on these findings, the 1000 mm/s was selected as the optimal LSS for SD247.

### 3.2. Effect of Composition on Cracking Tendency

**Figure 3:**
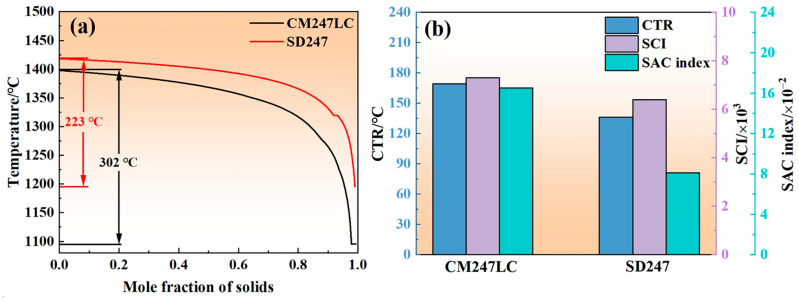
(**a**) Solidification paths of CM247LC and SD247 alloys in the Scheil–Gulliver module; (**b**) critical temperature range (CTR), solidification cracking index (SCI), and strain ageing cracking (SAC) index of CM247LC and SD247 alloys. Note: SD247 has lower Al, B, and Zr content and no Hf compared to CM247LC.

As noted in Section 3.1, compared to CM247LC, SD247 generally exhibits lower cracking susceptibility and enhanced LPBF processability. Thermodynamic calculations were conducted to explore the potential cracking-suppression mechanism of SD247 further. Figure 3a illustrates the solidification paths of both alloys using the Scheil–Gulliver module. The FR of SD247 (223 °C) is typically narrower than that of CM247LC (302 °C), which can be attributed to the removal of Hf and reductions in B and Zr, as reported by the literature [4,19,20]. The solidus temperature (T_s_) of SD247 is also higher than that of CM247LC. A lower T_s_ results in reduced liquid phase fluidity towards the end of solidification, making it more difficult for the liquid phase to flow and feed. This behaviour, combined with the wider FR and weaker fluidity, leads to the formation of voids in the interdendritic region towards the end of solidification. These voids are then susceptible to evolving into solidification cracks under thermal stress.

The phase precipitation behaviour during the nonequilibrium solidification process of CM247LC and SD247 alloys was further analysed. Low-melting-point (<1100 °C) borides and BCC/B2 phases occurred in CM247LC in the final stage of solidification [18]. These phases are prone to remelting during the thermal cycle in LPBF, forming liquid films and causing crack initiation. In contrast, due to the reduction in B in SD247, no borides were observed. Additionally, the removal of Hf significantly raised the solidus temperature (~1200 °C) of SD247, thereby reducing the liquid film formation [4].

If the alloy remains within the critical temperature range (CTR)—where the mole fraction of solids (*f*_s_) is between 0.9 and 0.99—for an extended period, the likelihood of cracking increases, and early-stage cracks are more prone to growth [27]. Figure 3b illustrates the CTR of both alloys. The CTR of SD247 was found to be 136 °C, which was narrower than that of CM247LC (169 °C). Following the method proposed by Kou [28], the solidification cracking index (SCI) was calculated to assess the solidification cracking susceptibility, with the *f*_s_ range set between 0.9 and 0.99:

(1) $$\mathrm{SCI}=\left|dT/d\left(f_{s}^{1/2}\right)\right|$$

where *f_s_* is a solid fraction and *T* is temperature; a higher SCI value indicates a greater tendency to crack. The SCI of CM247LC (~7300) was higher than that of SD247 (~6400), as displayed in Figure 3b.

The SAC index quantifies the susceptibility to strain-age cracking. Lower values indicate reduced risk. It can be calculated as follows:

(2) $$\mathrm{SAC}\mathrm{index}=dV_{f}^{\gamma }/dT,T\in \left[T_{\gamma }^{*},T_{soildus}\right]$$

where $V_{f}^{\gamma }$ is the mass fraction of the γ matrix and $T_{\gamma }^{*}$ is the temperature of 0.7 *T_solidus_*. The SAC index of CM247LC was found to be 0.165, whereas SD247 exhibited a significantly lower index of 0.081.

By integrating kinetics and thermodynamic databases, the γ′ phase precipitation behaviour during the thermal cycle of LPBF was then calculated. For CM247LC, the nucleation rate and number density of the γ′ phase at 1000 °C for 0.05 s were found to be 2.5 × 10^30^ m^−3^s^−1^ and 6.9 × 10^25^ m^−3^, respectively. In contrast, for SD247, these values were found to be 1.8 × 10^29^ m^−3^s^−1^ and 4.1 × 10^22^ m^−3^, respectively. These differences are primarily attributable to the reduction in Al and Hf (γ′ forming elements) content in SD247, further confirming the alloy’s lower susceptibility to SAC [9].

### 3.3. Microstructure of As-Built SD247

**Figure 4:**
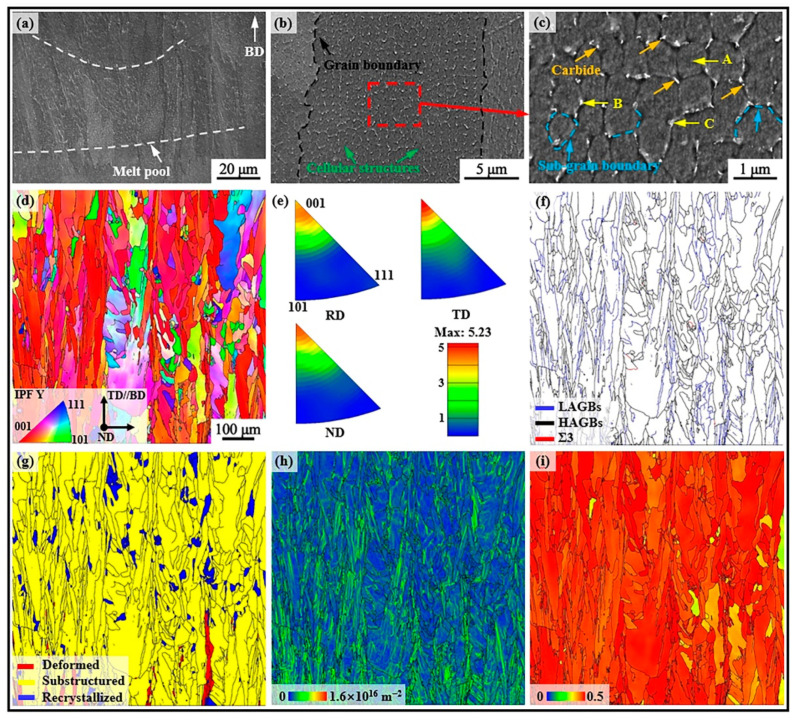
Microstructures of as-built SD247 alloy: (**a**) scanning electron microscope (SEM) images; (**b**) dendrite morphology; (**c**) high-magnification images of Figure 4b; (**d**) orientation imaging microscopy image; (**e**) inverse pole figure (IPF) map; (**f**) grain boundary (GB) map; (**g**) recrystallised component map; (**h**) geometrically necessary dislocation density map (the closer the colour is to the blue area, the lower the geometrically necessary dislocation density is); (**i**) Schmid factor map (the redder colour covers the grain, indicating that the grain is more easily deformed).

Figure 4 presents the microstructures of as-built SD247 alloy. At low magnification (Figure 4a), melt pools were clearly visible, while cellular structures were observed at higher magnification (Figure 4b). The shape of the cellular structure, which included both equiaxed and columnar forms, varied due to differences in heat dissipation direction. Further investigation revealed the presence of nanoscale irregularly shaped phases located at the sub-grain boundaries (sub-GBs). These phases were identified as (Ta, Ti)-rich MC-type carbides through EDS point scanning analysis, as detailed in Table 2. The orientation imaging microscopy image taken parallel to the building direction showed numerous columnar grains, with the majority exhibiting a preferred orientation of <001>//building direction. This observation was further confirmed by inverse pole figure (IPF) analysis. The maximum texture density value from the IPF analysis was found to be 5.23. The average grain diameter was measured to be 26.2 μM, with an average aspect ratio of 4.6, as determined using HKL Channel 5 software. Compared to casted Ni-based alloys, the grains in LPBF-fabricated SD247 achieved a significant refinement, contributing to the improvement in strength and ductility. LPBF-fabricated alloys also avoid completely shrinkage cavity defects in casting alloys. All of these are beneficial in improving the reliability of Ni-based alloy components during industrial applications.

Figure 4f shows the grain boundary (GB) distribution map of SD247. The blue lines represent low-angle grain boundaries (LAGBs), with a misorientation between 2° and 15°, while the black lines indicate high-angle grain boundaries (HAGBs), with a misorientation of greater than 15°. The fraction of HAGBs was found to be notably higher than that of LAGBs, with the respective proportions being 65.7% and 34.3%. The red lines denote a special class of HAGBs known as Σ3 GBs, among the most common types of coincidence site lattice (CSL) GBs found in Ni-based alloys [29]. CSL GBs are generally characterised by lower boundary energy and greater structural stability. Previous studies have confirmed that a higher fraction of CSL GBs in an alloy improves several properties, including plasticity and fatigue resistance [15,18]. Data analysis revealed that the fraction of Σ3 GBs in SD247 was only 0.2%. Figure 4g presents the recrystallised component map of SD247. Based on the average misorientation angle within the grains, the grains were categorised into three types: deformed, sub-structured, and recrystallised, colour-coded as red, yellow, and blue, respectively. Only 2.3% of the grains were classified as deformed, while 5.8% were recrystallised and 91.9% were sub-structured.

In general, the abnormal metallurgical characteristics inherent in the LPBF process induce higher residual stresses in the alloy, leading to crystal deformation and orientation gradients within the grains. To accommodate these gradients, geometrically necessary dislocations were generated, with their density serving as an indirect indicator of the stress level [18]. The geometrically necessary dislocation density map for SD247 is shown in Figure 4h. Further analysis revealed that the average geometrically necessary dislocation density for SD247 was 2.5 × 10^15^ m^−2^. The Schmid factor for the {111}〈101〉 slip system was calculated for grains with the loading direction (*x*-axis) perpendicular to the building direction, as shown in Figure 4i. The Schmid factor provides a measure of the difficulty of deformation under loading. Most grains are highlighted in red, indicating a high Schmid factor. Generally, grains with higher Schmid factor values are more prone to deformation, meaning they have a softer orientation [30]. The statistical analysis indicated that the average Schmid factor for as-built SD247 was approximately 0.44. Grains with Schmid factor values in the range of 0.45–0.5 are typically considered easily deformable. During loading, plastic deformation should ideally occur in these grains, which will contribute to a reduction in YS. The fraction of such grains in as-built SD247 was found to be 74.1%.

### 3.4. Microstructure of Heat Treatment SD247

**Figure 5:**
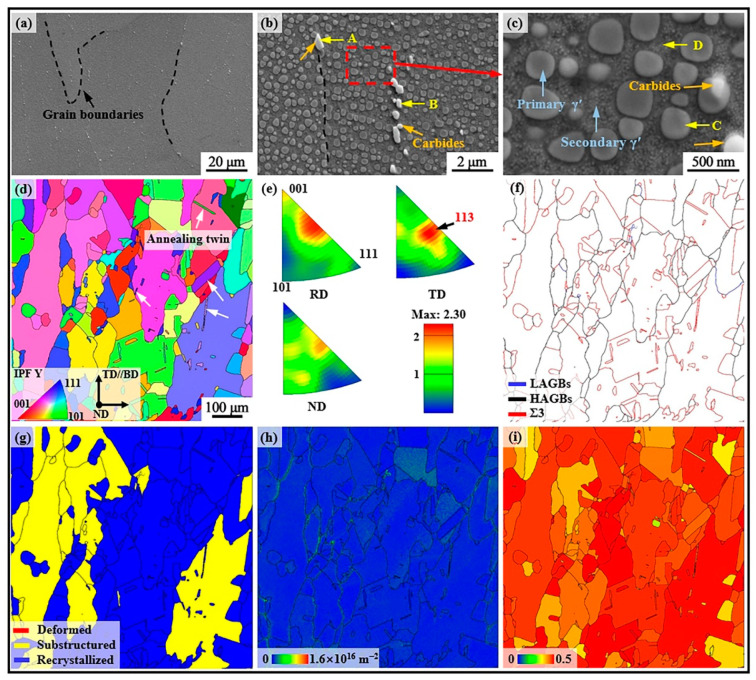
Microstructures of heat treatment SD247 alloy: (**a**,**b**) SEM images; (**c**) higher-magnification image of Figure 5b; (**d**) orientation imaging microscopy image; (**e**) IPF map; (**f**) GB map; (**g**) recrystallised component map; (**h**) geometrically necessary dislocation density map; (**i**) Schmid factor map.

The microstructure of heat-treated SD247 was found to exhibit significant differences compared to that of as-built specimens, as shown in Figure 5a–c. Notably, no microcracks were observed after heat treatment. The melt pool boundaries disappeared, and dispersed white blocky phases were observed both at the GBs and within the grains. These phases were likely associated with nanoscale granular carbides present at the sub-GBs of the as-built alloy. The carbides did not dissolve during heat treatment but instead merged and grew due to their high melting points. High-magnification images revealed that the average size of the block phases was approximately 130 μM. The EDS point scanning results, presented in Table 3, confirmed that these phases were MC-type carbides enriched in (Ta, Ti). A significant number of near-square γ′ phases, with an average size of about 306 nm, were also observed within the γ matrix. These phases, which precipitated during the first-stage ageing treatment, are referred to as primary γ′ phases. As shown in Figure 5c, a bimodal distribution of γ′ phases was observed, with the fine spherical γ′ phases (averaging about 25 nm) found between the primary γ′ phases. These fine phases are referred to as secondary γ′ phases since they precipitated during the second-stage ageing treatment. The lower ageing temperature resulted in reduced element diffusion rates, thereby reducing the size of the γ′ phases [31].

The orientation imaging microscopy image of heat-treated SD247 shown in Figure 5d revealed significant changes in the grain structure. The <001>//building direction preferred orientation observed in the as-built alloy disappeared after heat treatment, with the grains exhibiting a more random orientation. According to the IPF of heat-treated SD247, a weak <113>//building direction orientation was detected. Heat-treated also induced the formation of numerous annealing twins, typical in Ni-based alloys, due to their low stacking fault energy [29]. Compared to the as-built SD247, the grain size of heat-treated SD247 increased significantly from 26.2 μM to 62.8 μM (excluding twins), while the aspect ratio of the grains decreased from 4.6 to 2.5, a reduction of 45.7%. This finding demonstrates that heat treatment had a substantial effect on both grain size and morphology. As shown in Figure 5f, LAGBs almost completely disappeared, with their fraction reducing to only 1.4%, while the fraction of HAGBs increased to 98.6%. In general, the larger the orientation difference between grains across HAGBs, the more energy is required for intergranular crack propagation; the higher fraction of HAGBs, thus, is beneficial for improving the alloy’s strength. Orientation imaging microscopy image analysis of heat-treated SD247 also revealed a 60°//<111> orientation relationship between the annealing twins and the parent phase grains. Twin boundaries in Ni-based alloys are typically equivalent to Σ3 CSL GBs. These can enhance an alloy’s strength without significantly reducing elongation, both at room and elevated temperatures [32]. After heat treatment, the fraction of Σ3 GBs increased sharply to 56.4%.

Figure 5g presents the recrystallised component map of heat-treated SD247. The ‘deformed’ grains were completely eliminated, and the fraction of ‘recrystallised’ grains increased significantly from 5.8% to 60.8%, indicating that heat treatment had effectively promoted the recrystallisation process. As shown in the geometrically necessary dislocation density map (Figure 5h), the geometrically necessary dislocation density decreased substantially. Further calculations revealed that the average geometrically necessary dislocation density of heat-treated SD247 decreased by 62.1%, from 2.5 × 10^15^ m^−2^ in the as-built state to 9.5 × 10^14^ m^−2^ in the heat-treated alloy. This reduction in geometrically necessary dislocation density suggests that heat treatment facilitated the elimination of the orientation gradient within the grains and relieved the residual stresses induced by LPBF. These changes are attributable to the decreased brittleness and improved deformation capability of the alloy.

The Schmid factor map of heat-treated SD247 is shown in Figure 5i. The fraction of grains with a high Schmid factor (the crimson colour) decreased significantly, while the fractions of grains with intermediate Schmid factor values (yellow and orange) increased markedly. A closer examination revealed that the average Schmid factor of heat-treated SD247 increased to 0.46, compared to 0.44 for the as-built alloy. The fraction of grains with a Schmid factor between 0.45 and 0.5 decreased from 74.1% to 64.6%, representing a 12.8% reduction. This reduction in the fraction of easily deformed grains following heat treatment contributed to the enhanced strength of the alloy.

### 3.5. Mechanical Properties of the Specimens

**Figure 6:**
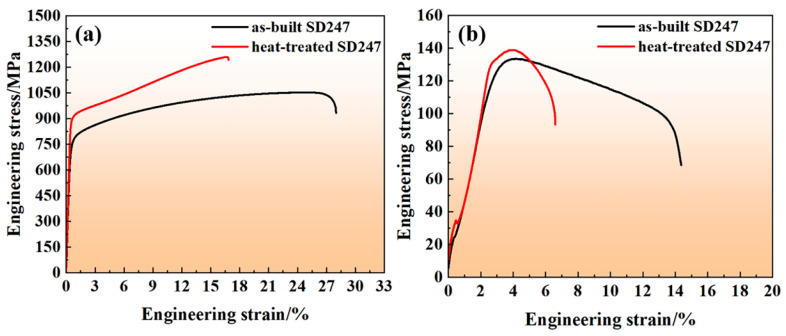
Engineering stress–strain curves of SD247: (**a**) at room temperature; (**b**) at elevated temperature.

The microhardness values of both as-built and heat-treated SD247 were measured by selecting 10 random positions on the polished surface of each sample. The microhardness values presented are averages of these 10 measurements. The microhardness of as-built SD247 was 305.5 HV, while heat-treated SD247 exhibited a microhardness of 397.4 HV, marking a 30.1% increase. Figure 6 presents the engineering stress–strain curves of SD247 at room temperature and elevated temperature, with the corresponding tensile test results summarised in Table 4. At room temperature, as-built SD247 exhibited a UTS of 1033 ± 20 MPa, a YS of 740 ± 17 MPa, and an EL of 22.5 ± 2.2%. Following heat treatment, SD247 showed significant improvements in both UTS and YS, with values of 1241 ± 18 MPa and 872 ± 15 MPa, respectively, although EL decreased to 15.9 ± 0.6%. The observed changes in mechanical properties post-heat treatment were likely due to the precipitation of the γ′ phase. The presence of the γ′ phase also created numerous stress concentration sites, which facilitated crack initiation and ultimately led to a reduction in ductility.

At elevated temperatures, as-built SD247 exhibited a UTS of 128 ± 3 MPa and an EL of 11.8 ± 0.6%. In contrast, heat-treated SD247 showed a slightly higher UTS of 135 ± 4 MPa but a reduced EL of 3.9 ± 0.5%. The relatively low dissolution temperature of the γ′ phase (1108 °C, Figure 1d) resulted in significant γ′ phase dissolution at 1100 °C, diminishing the precipitation strengthening effect. This behaviour led to more modest improvements in UTS after heat treatment. In general, under high-temperature conditions, GBs become weaker points that are more susceptible to deformation. Due to the reduction in GB-strengthening elements (B, Zr, and Hf), GBs of heat-treated SD247 were found to be relatively clean (Figure 5b), with very few blocky carbides, which were insufficient to effectively hinder GB sliding. The sharp edges of these carbides induced stress concentrations in GBs, however, which facilitated crack initiation and promoted fracturing. In contrast, as-built SD247 has an ultra-fine cellular structure, which can share the stress during loading [30]. Fine carbides in sub-GBs effectively impede dislocation movement without causing significant stress concentrations. Such behaviour contributed to a substantially reduced EL at elevated temperatures for heat-treated SD247. This may not be conducive to engineering applications. Hence, addressing the trade-off in mechanical properties at elevated temperatures will be the key mission in our future work.

Table 5 shows the comparative analysis of mechanical properties at room temperature between heat-treated SD247 and other aerospace alloys with high γ′ phase fraction [33,34,35]. Heat-treated SD247 exhibited the highest UTS and EL. Based on the microstructure analysis, the remarkably improved properties can be attributed to grain refinement due to rapid cooling speed. This suggests that heat-treated SD247 offers excellent strength–ductility balance. Moreover, compared to CM247LC, SD247 has no Hf (an expensive element), demonstrating a lower cost. Hence, SD247 shows great potential in LPBF fabricating aerospace hot-end components with complex-shaped structures, which are challenging to fabricate via conventional processes.

### 3.6. Possible Strengthening Mechanisms

The following primary strengthening mechanisms can improve the YS at room temperature: (i) GB strengthening (*σ_GB_*), (ii) solid solution strengthening (*σ_SS_*), (iii) dislocation strengthening (*σ_DIS_*), and (iv) precipitation strengthening (*σ_PRE_*). The remarkable improvement in post-heat treatment YS at room temperature can thus be calculated by the following equation:

(3) $$\sigma_{y}=\sigma_{0}+\sigma_{GB}+\sigma_{SS}+\sigma_{DIS}+\sigma_{PRE}$$

where *σ_y_* is the YS of the alloy and σ_0_ is the intrinsic strength, which is 52.5 MPa [36]. The grain refinement plays an important role in strengthening because of the Hall–Petch relationship. Equation (4) may be used to express the YS improvement by GB enhancement [36]:

(4) $$\sigma_{GB}=Kd^{-1/2}$$

where K is the Hall–Petch coefficient, which has been determined to be 750 MPa μM^1/2^ for Ni-based alloys [37], and *d* is the average grain diameter. Since the cellular structure significantly hinders dislocation movement, the average diameter of the cellular structure is *d* for the as-built alloy [38]. For heat-treated SD247, *d* may be detected from EBSD data. On this basis, *σ_GB_* was calculated in this study as 348.4 MPa (as-built SD247) and 136.1 MPa (heat-treated SD247), respectively.

The contribution of solid solution strengthening to YS is expressed by the following equation [23]:

(5) $$\sigma_{SS}={\left(\sum_{i}k_{i}^{2}c_{i}\right)}^{1/2}$$

where *k_i_* is the strengthening constant of each solute element, which was obtained from the literature [37], and *c_i_* is the atom fraction of elements, which was detected by EDS point scanning. As shown in Table 3 and Table 4, the content of solid solution elements in the matrix was not markedly different, so the contribution of solid solution strengthening was not included in the specific calculation.

The contribution of dislocation strengthening to YS may be calculated by Taylor’s law, as illustrated in Equation (6) [23]:

(6) $$\sigma_{DIS}=\alpha \mathrm{MbG}\rho^{1/2}$$

where α is a constant for FCC alloys (0.2), M is the Taylor factor (3.06), b is the magnitude of the Burgers vector (0.25 nm), G is the shear modulus of the alloys, and *ρ* is the average geometrically necessary dislocation density [23]. Accordingly, the YS-contributed dislocation strengthening of heat-treated SD247 will be −174.2 MPa compared to that of as-built SD247.

The precipitation strengthening mechanism may be categorised into dislocation bypassing and dislocation cutting, depending on the particle diameter. When the particle diameter exceeds 200 nm, the dislocation bypassing mechanism becomes predominant. Conversely, when the particle diameter is smaller than 200 nm, the dislocation cutting mechanism dominates, as reported by Fang et al. [39]. The dislocation bypassing mechanism may be expressed as the following equations:

(7) σoro=3Gb2L

(8) L=2π3f1/2r

where *σ_oro_* is the contribution of the dislocation bypassing mechanism to YS, *L* is the average precipitation spacing, *r* is the average particle radius, and *f* is the volume particle fraction. Note that the dislocation shearing mechanism is divided into a strong-pair coupling mechanism (r > 20 nm) and weak-pair coupling (r < 20 nm). Since the r of the particle in this study was greater than 20 nm, the strong-pair coupling mechanism played a larger role. Hence, the dislocation shearing mechanism may be expressed as follows:

(9) $$\sigma_{strong}=\sqrt{\frac{3}{2}}\left(\frac{\mathrm{Gb}}{r}\right)\frac{f^{1/2}}{\pi^{3/2}}{\left(\frac{2{\pi \gamma }_{\mathrm{APB}}r}{{\mathrm{Gb}}^{2}}-1\right)}^{1/2}$$

where *σ_strong_* is the YS improvement by the dislocation shearing mechanism, and γ_APB_ is the anti-phase boundary (APB) energy, which was determined to be 0.178 J/m^2^ [39]. Because *σ_strong_* may not be accurate due to the effect of GB strengthening and solid solution strengthening, a constant of 100 MPa/*f*^1/2^ was added to the model as compensation [40].

To summarise, the contribution of precipitation strengthening to the YS of heat-treated SD247 was found to be 486.7 MPa. Precipitation strengthening thus was the main strengthening pattern for heat-treated SD247, rather than fine grain strengthening, dislocation strengthening, or solid solution strengthening.

**Figure 7:**
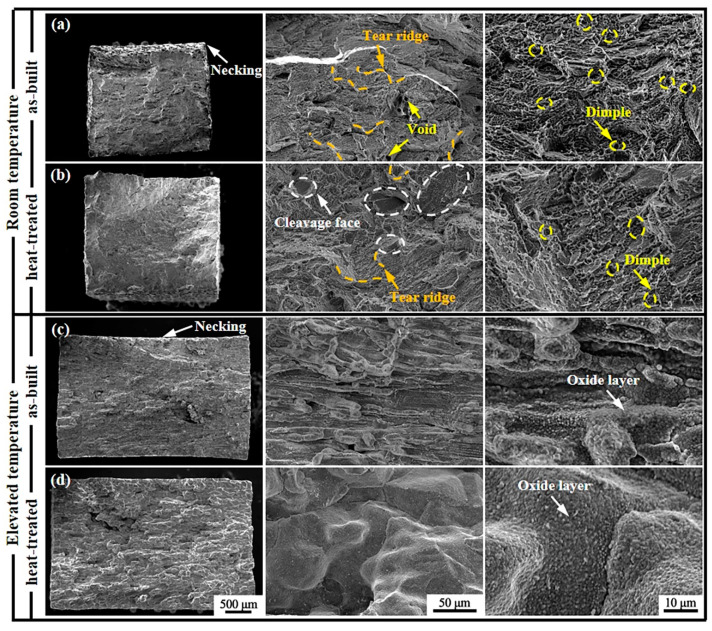
SEM images of the fracture surfaces of SD247 at room temperature (**a**,**b**) and elevated temperature (**c**,**d**).

Figure 7 presents the SEM images of the fracture surfaces of SD247 at room temperature and elevated temperature. At room temperature, the fracture surface of as-built SD247 exhibited clear necking and an undulating texture, with noticeable tear ridges and numerous dimples, indicating excellent ductility. In contrast, the necking in heat-treated SD247 was significantly reduced, although the fracture surface remained undulating. At higher magnification, a few cleavage faces and tear ridges were observed, and the fracture surface showed shallow and sparse dimples, suggesting that a quasi-cleavage fracture mode and poorer ductility existed compared to as-built SD247. These observations were consistent with the tensile test results.

Figure 7c,d show the fracture surfaces of SD247 at the elevated temperature. Necking was only observed in as-built SD247, not in heat-treated SD247, further indicating the superior ductility of the former. The fracture surface of heat-treated SD247 was also noticeably flatter than that of as-built SD247. However, since the tensile tests at the elevated temperature were conducted in an unprotected atmospheric environment, an oxide layer was present on all fracture surfaces, limiting further detailed analysis.

## 4. Conclusions

This study has proposed a novel precipitation-strengthened crack-free Ni-based alloy (SD247) for LPBF and systematically investigated the LPBF processability, microstructure, and mechanical properties of SD247. The primary conclusions are as follows:

(1) Alloying element modification in SD247 successfully addresses the high cracking sensitivity issue in LPBF-fabricated high γ′ phase-strengthened nickel-based superalloys. As-built exhibited a typical cellular structure, characterised by a few ultra-fine carbides at the sub-GBs, along with pronounced columnar grains.
(2) After heat treatment, the cellular structure in SD247 completely disappeared. A bimodal distribution of the γ′ phase was observed in the matrix, along with limited blocky MC-type carbides. The formation of numerous annealing twins was noted, and the fraction of grains with higher Schmid factor decreased, suggesting improvements in the alloy’s mechanical properties.
(3) Precipitation strengthening was identified as the primary factor responsible for post-heat treatment improvements in YS. Heat-treated SD247 demonstrated a significant enhancement in both UTS (1241 ± 18 MPa) and EL (15.9 ± 0.6%), resulting in an improved strength–ductility balance.
(4) LPBF-fabricated SD247 exhibits better mechanical properties and cracking resistance, making it well suited for aerospace hot-end components with complex geometries. The removal of Hf in SD247 makes it more cost-effective than alloys like CM247LC, improving its potential for large-scale production. Its superior mechanical properties, coupled with low cost, make it a promising alternative to traditional Ni-based alloys during LPBF.
(5) Further work should focus on addressing poor ductility at elevated temperature conditions from GB morphology and grain structure, exploring its performance in long-term operational conditions, and conducting the fabrication of complex components to achieve industrial implementation.

## Figures and Tables

**Table 1:** Chemical composition of CM247LC and SD247 powders (wt.%).

Alloy	Cr	Co	W	Mo	Al	Ti	Ta	C	B	Zr	Hf
CM247LC	8.5	9.5	9.4	0.59	5.7	0.86	2.9	0.08	0.02	0.02	1.4
SD247	8.6	9.8	9.6	0.58	4.2	0.78	3.0	0.06	0.005	0.005	–

**Table 2:** Results of energy-dispersive X-ray spectroscopy (EDS) point analysis for the area marked in Figure 4c (wt.%).

Point	Cr	Co	W	Mo	Al	Ti	Ta	C	B	Zr
A	7.45	9.35	10.10	1.51	3.47	0.61	2.57	–	–	–
B	8.72	8.16	10.45	2.07	3.40	1.96	6.82	7.93	–	0.11
C	8.45	8.08	9.70	2.19	3.56	1.51	4.79	7.89	–	0.09

**Table 3:** Results of EDS point analysis for the area marked in Figure 5b,c (wt.%).

Point	Cr	Co	W	Mo	Al	Ti	Ta	C	B	Zr
A	1.21	0.76	5.50	0.90	0.38	11.94	58.03	10.88	/	/
B	3.53	2.79	8.44	1.07	1.39	9.13	46.38	13.40	/	0.03
C	3.69	4.52	8.30	0.73	5.43	0.89	5.45	/	/	0.08
D	8.37	9.43	9.88	1.13	3.28	0.26	2.90	/	/	/

**Table 4:** Tensile properties of SD247 at room temperature and elevated temperature.

Condition	Room Temperature			Elevated Temperature	
	UTS/MPa	YS/MPa	EL/%	UTS/MPa	EL/%
As-built	1033 ± 20	740 ± 17	22.5 ± 2.2	128 ± 3	11.8 ± 0.6
Heat-treated	1241 ± 18	872 ± 15	15.9 ± 0.6	135 ± 4	3.9 ± 0.5

**Table 5:** Comparative analysis of mechanical properties at room temperature between heat-treated SD247 and other aerospace alloys with high γ′ phase fraction.

	Fabrication Method	UTS/MPa	EL/%	Reference
SD247	LPBF	1241	15.9	This work
IN738LC	Vacuum investment casting	1034	7	[33]
IN738	Vacuum investment casting	1103	3	[33]
René 80	Vacuum investment casting	1034	5	[34]
MAR M247	Investment casting	980	6	[35]

## Data Availability

The original contributions presented in the study are included in the article, further inquiries can be directed to the corresponding authors.
